# The Mixing Counterion Effect on DNA Compaction and Charge Neutralization at Low Ionic Strength

**DOI:** 10.3390/polym10030244

**Published:** 2018-02-28

**Authors:** Yanwei Wang, Ruxia Wang, Tianyong Gao, Guangcan Yang

**Affiliations:** College of Mathematical, Physics and Electronic Information Engineering, Wenzhou University, Wenzhou 325035, China; wangyw@wzu.edu.cn (Y.W.); rxwangwz@163.com (R.W.); wzugty@163.com (T.G.)

**Keywords:** DNA compaction, charge neutralization, counterion, condensing forces

## Abstract

DNA compaction and charge neutralization in a mixing counterion solution involves competitive and cooperative electrostatic binding, and sometimes counterion complexation. At normal ionic strength, it has been found that the charge neutralization of DNA by the multivalent counterion is suppressed when being added extra mono- and di-valent counterions. Here, we explore the effect mixing counterion on DNA compaction and charge neutralization under the condition of low ionic strength. Being quite different from normal ionic strength, the electrophoretic mobility of DNA in multivalent counterion solution (octalysine, spermine) increases the presence of mono- and di-valent cations, such as sodium and magnesium ions. It means that the charge neutralization of DNA by the multivalent counterion is promoted rather than suppressed when introducing extra mono- and di-valent counterions into solution. This conclusion is also supported by the measurement of condensing and unraveling forces of DNA condensates under the same condition by single molecular magnetic tweezers. This mixing effect can be attributed to the cooperative electrostatic binding of counterions to DNA when the concentration of counterions in solution is below a critical concentration.

## 1. Introduction

DNA is an extraordinarily important biological macromolecule storing genetic information and carrying instructions for protein synthesis. Due to the highly charged nature of nucleic acids, they are constantly surrounded by counterions to neutralize most of their charges to reduce the electrostatic repulsion between segments of DNA, so they can be condensed into compact, orderly structures [1]. The understanding of the DNA compaction process is not only important for the study of fundamental biological processes such as chromosome compacting, but it also has potential clinical applications such as the development of new vehicles for gene therapy [2,3,4]. DNA compaction process is related to counterion association [5,6,7,8,9], charge inversion [10,11,12] and like-charged attraction [13,14,15,16,17,18]; it involves many factors such as cation type, counterion concentration, temperature, dielectric properties of solvent and pH in solution [19,20,21,22,23,24,25,26,27,28,29,30,31,32].

Dynamic light scattering (DLS) [33] enables the characterization of sizes and electrokinetic properties of colloids, polymers and macromolecules. Thus, it is a feasible tool to study the interaction between DNA and counterions [34,35]. Meanwhile, some newly developed single molecule approaches make it possible for us to get more insights into the interaction. For example, magnetic tweezers (MT) are versatile tools for the study of a single biological macromolecule under applied force, not only being able to measure the exerting force but also the torque of tethering molecules [10,36,37]. The atomic force microscopy (AFM) can not only provide conformal and morphological information, but also is suitable to study inter- and intra-molecular interaction forces in biological macromolecules [12,38,39,40].

In physiological ionic solutions and cells, there are various cations and anions of different size and charge, such as Na^+^, K^+^, Mg^2+^, Ca^2+^, and Cl^−^. In classical Manning-Oosawa condensation theory, the fraction of counterions condensing on uniformly charged rod depends only on the valence of counterions, but is independent of other factors, such as type of ions, pH and ionic strength in solution [41]. When many types of counterions exist in solution, the electrostatic interaction between DNA and counterions becomes much more complicated than the case of single counterion [42,43,44,45,46,47]. It has been shown that increasing monovalent salt concentration hinders charge inversion by multivalent ions [10,48]. In a mixed ion solution that contains a mild amount of tetravalent cations, we found that charge neutralization of DNA is suppressed by mono- and di-valent cations but promoted by trivalent cations [21].

In the present study, we explore the competition effect of mixing counterions on DNA compaction and charge neutralization under the condition of low ionic strength. DLS and MT are used to investigate the interaction between DNA and multivalent counterions (polylysine and spermine) when additional mono- or di-valent ions are present in solution. The influence of additional cations is characterized by size and electrophoretic mobility of DNA condensates through DLS, while the force spectroscopy can be obtained by pulling DNA complexes in MT. In a low ionic strength condition, the electrophoretic mobility of DNA in multivalent counterion solution increases in presence of mono- and di-valent cations, which is contrary to the case of normal ionic strength. It means that the charge neutralization of DNA by multivalent counterions is promoted rather than suppressed when adding extra mono- and di-valent counterions. Meanwhile, we measure condensing and unraveling forces of DNA condensates induced by mixing counterions at low ionic strength by MT. The force spectroscopic data is consistent with the result of the promotion of electrophoretic mobility.

## 2. Experimental Procedures

### 2.1. Materials

Double stranded λ-phage DNA (48,502 bp) for MT and electrophoretic-mobility (EM, μ) measurements was purchased from New England Biolabs (Beijing, China) LTD. and did not go through purification before using. The stoke solution of DNA is 1× TE buffer (10 mM Tris-HCl (pH = 8.0) and 1 mM EDTA) and the final DNA concentration is 500 ng·μL^−1^. We used 4 different types of multivalent counterions for the experiment of DNA compaction and charge neutralization to make sure that the results are consistent and universal for DNA system. Among the agents of cations, 4-poly-l-lysine (K4), 8-poly-l-lysine (K8) were ordered from Qiang Yao Biology Limited Company (Shanghai, China). The other chemical and biochemical agents (such as various salts, bovine serum albumin (BSA), and hydroxylmethylaminoethane (TRIS)) were purchased from Sigma-Aldrich (St. Louis, MO, USA) and used as received. Phosphate buffer saline (PBS) was used in our sample preparation, containing 10 mM phosphate, 140 mM NaCl at pH 8.0. Electrophoretic mobility of DNA at various concentration of mixing counterions was measured in a 1 mM TRIS buffer at pH 8.0. All solutions were prepared with deionized water of resistivity 18.2 MΩ·cm purified through the Milli-Q system (Millipore Corporation, Bedford, MA, USA). We repeats all measurements at least 3 times to obtain consistent results, and the standard deviation was calculated accordingly.

### 2.2. Methods

EM measurement was carried out by using DLS device of Malvern Zetasizer nano ZS90 (Malvern Instruments Limited Company, Malvern, UK) equipped its patented M3-PALS technique, in which a He-Ne laser (λ = 633 nm) is applied and an avalanche photodiode is used to detect the scattering light. DNA was diluted to a concentration of 1 ng·μL^−1^ in a TRIS buffer in dynamic light scattering measurement, by adding various counterions with different concentration in solution. Before measurement, 5 min are needed for incubation at room temperature. In the measurement of electrophoretic mobility, a sample of 1 mL of DNA solution was pipetted in the folded capillary cell. During the process of measurement, sample cell was kept at a constant temperature of 25 °C.

**Figure 1:**
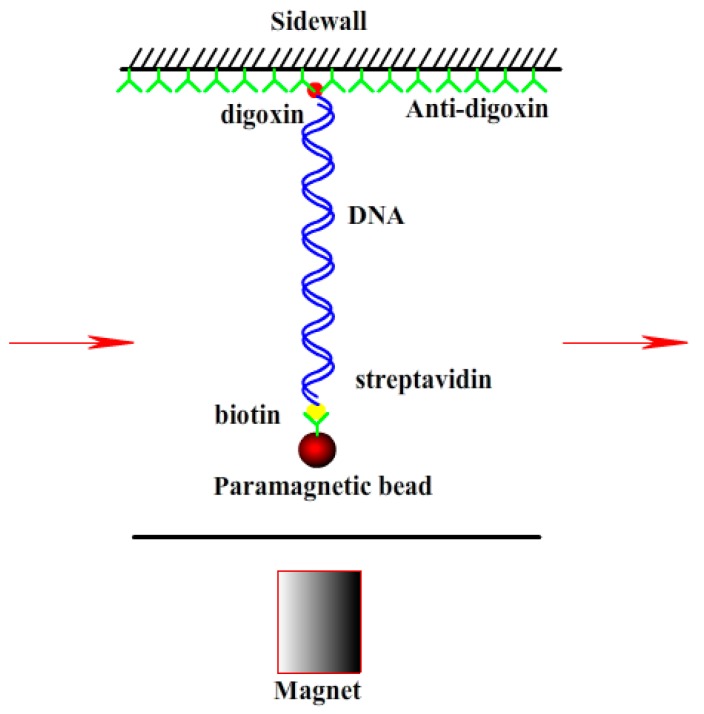
Sidewall-DNA-bead structure for single molecule magnetic tweezers (MT) experiment.

A transverse MT was used to obtain the force spectroscopy of DNA in counterion solution. The detail of setup is as described before [19,37]. In brief, the flow chamber with a polished sidewall was dealt with anti-digoxygenin at first and then was rinsed with PBS containing 5 mg·mL^−1^ bovine serum albumin (BSA) at pH 8.0. DNA-bead constructs were then flushed into the cell, then a side wall-DNA-paramagnetic bead structure was formed, as shown in Figure 1. The extension of DNA is approximately the distance between the bead and the surface of sidewall. The applied force to the bead is related with Brownian motions of the microsphere and can be calculated accordingly. The DNA extension was determined by a tracking algorithm of fast Fourier transform-based correlation techniques. The shrinking or pulling of single DNA chain can be monitored by measuring the DNA extension in time while lowering or increasing the tethering force by moving the magnet slowly. Before measurements, the BSA in buffer has to be removed by rinsing with 1 mL PBS to avoid the interference of BSA to the process of DNA compaction. After finding out a single suspending lambda-DNA, the bead was pulled to its maximal displacement to the sidewall. Then mixing counterions at different concentrations were flushed to the flow cell and incubated for 10 min, then the elastic response of DNA as a function a time was recorded and analyzed at different forces. The speed for the inflow of the DNA constructs or the counterion solution into the cell is 10 μL·min^−1^. The condensing force (*F*_C_) is the force when the first step-like shrinking in DNA extension-time curve occurred. When DNA is compacted, the magnetic bead is close to the sidewall to form a compact structure. It can be unraveled by moving the magnet toward the sidewall. The pulling force disassembling the DNA condensate is defined as unraveling force (*F*_U_).

## 3. Results

### 3.1. Suppression and Promotion of DNA Charge Inversion by Mixing Multivalent and Monovalent Counterions

The electrophoretic mobility of DNA complex, μ, depends linearly on its total charge including the bare charge of DNA and the charge of counterions at its surface [49,50]. It reflects the extent of charge neutralization of DNA by counterions in solution.

**Figure 2:**
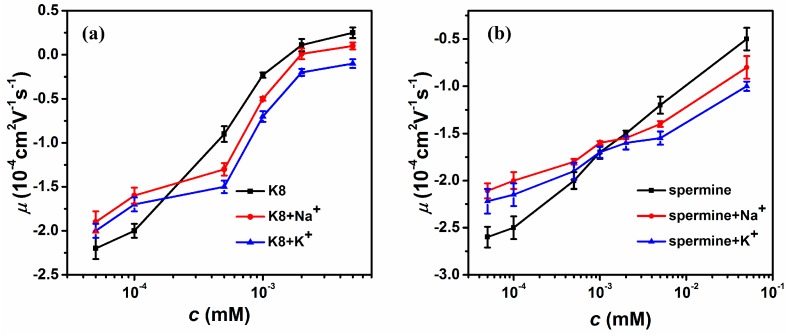
Electrophoretic mobility μ of condensed DNA as a function of the multivalent ions in a buffer containing monovalent ions (3 mM Na^+^, 3 mM K^+^). (**a**) The data for K8, K8-Na^+^ solution and K8-K^+^ solution (black, red, blue). (**b**) The data for spermine, spermine-Na^+^ solution and spermine-K^+^ solution (black, red, blue). Each data point is the average of three consecutive measurements with the corresponding standard deviation as the error.

Figure 2 shows the measured mobility of condensed DNA as a function of the concentration of 8-poly-l-lysine (K8) and spermine in various cation solutions. The DNA electrophoresis mobility with 1 mM TRIS buffer containing K8 mixing with 3 mM monovalent sodium and potassium ions are shown in Figure 2a. In general, we can see that the mobility of DNA goes up from negative to positive values with increasing K8 concentration. However, when additional sodium or potassium was added to the solution, the mobility was changed accordingly. An apparent feature which can be noted in the figure is the crossing of mobility curves corresponding to single and mixing counterions. When the concentration of K8 is less than about 0.0002 mM, the DNA mobility shifts to a less negative value after mixing sodium or potassium ions in solution. In other words, the DNA charge neutralization is promoted by mixing the K8 and monovalent ions (3 mM). 

After the crossing point, the scenario is switched to the opposite side. When the concentration of K8 is more than about 0.0002 mM, the DNA mobility shifts to a small value after mixing sodium or potassium ions in solution. The DNA charge compensation is suppressed by adding monovalent cations. It resumes to the case of normal ionic strength (>0.0064 for K8) we explored before [10,21,48]. For example, when the concentration of K8 is 0.0001 mM, the value of μ was −2 (in units of 10^−4^ cm^2^ V^−1^ s^−1^, the same unit is assumed for mobility in the following unless otherwise stated). When the monovalent ion (3 mM) was added to K8-DNA solution, the value of μ climbed slightly up to −1.6 (Na^+^) and −1.7 (K^+^), respectively. We can see that the change of DNA electrophoresis mobility by adding Na^+^ is slightly bigger than K^+^, implying that the effect of charge neutralization promotion by sodium ion is more obvious. After crossing the critical concentration of K8, the promotion becomes some suppressive. More specifically, when K8 is present alone in solution and its concentration is 0.0005 mM, the value of μ is −0.9. When the monovalent ion (3 mM) was added to K8-DNA solution, the value of μ descends to −1.3 (NaCl) and −1.5 (KCl), respectively. Similarly, we can see that the change of DNA electrophoresis mobility by adding K^+^ is more significant in the case of Na^+^, corresponding to more obvious suppression of charge neutralization. The result is also valid for the other multivalent counterion spermine. Figure 2b shows the DNA electrophoresis mobility with 1 mM TRIS buffer containing spermine with or without mixing with sodium chloride or potassium chloride in solution. In the same way, the mobility of DNA goes up with increasing spermine concentration. There is also a crossing concentration of spermine of 0.001 mM. Before the crossing value, the DNA mobility shifts to a less negative after mixing sodium or potassium ions in solution. After the crossing concentration, the suppression of charge neutralization can be observed when sodium chloride or potassium chloride were added to the spermine-DNA solution.

**Figure 3:**
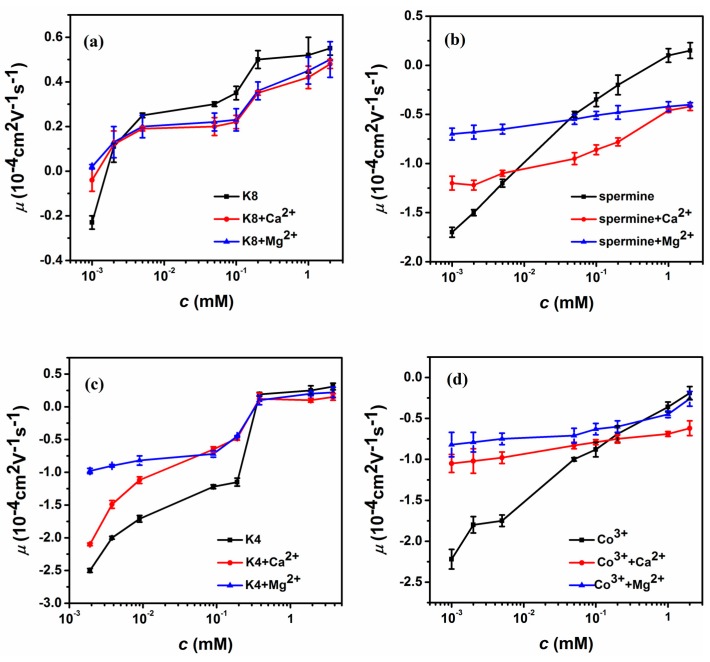
Electrophoretic mobility μ of condensed DNA as a function of the multivalent ions containing di-valent ions (3 mM Ca^2+^, 3 mM Mg^2+^) in 1 mM TRIS. (**a**) The EM as a function of K8, K8-Ca^2+^, K8-Mg^2+^. (**b**) The EM as a function of spermine, spermine-Ca^2+^, spermine-Mg^2+^. (**c**) The EM as a function of K4, K4-Ca^2+^, K4-Mg^2+^. (**d**) The EM as a function of Cobalt hexamine, Cobalt hexamine-Ca^2+^, Cobalt hexamine-Mg^2+^. Each data point is the average of three consecutive measurements with the corresponding standard deviation as the error.

To find out whether the promotion or suppression of charge compensation of DNA is a universal phenomenon, we further explore the mixing effect of di-valent cations. The result of electrophoretic light scattering is shown in Figure 3, which is consistent with the case of monovalent ions. In Figure 3a, we plot the electrophoretic mobility of DNA versus the concentration of K8 with or without di-valent ions (3 mM Ca^2+^ and Mg^2+^) in solution. Because of the highly charged feature of K8, the DNA electrophoresis mobility ascends rapidly from negative to positive with increasing concentration of K8, implying net charge inversion of the DNA-K8 complex. Similar to the case of mixing monovalent ions, the current electrophoretic mobility curves shift upwards or the charge neutralization of DNA is promoted when the concentration of K8 is smaller than 0.002 mM by adding di-valent counterions to the solution. Comparing the curves of two di-valent cations, we can see that the shifts of DNA electrophoresis mobility are almost the same though the influence of adding MgCl_2_ is slightly bigger than CaCl_2_. When the concentration of K8 crosses the critical value, 0.002 mM, the scenario of suppression is recovered as expected by adding di-valent counterions to the solutions. In this case, the DNA’s mobility curves shift downward, corresponding to the charge compensation or inversion of DNA being suppressed. We can see that the suppressing effect of charge inversion induced by Ca^2+^ is more significant by Mg^2+^. We tried many multivalent cations to test the promotion and suppression effect by mixing di-valent ions to ensure consistency. Figure 3b shows the case of tetravalent spermine, in which DNA charge inversion also happens when the concentration of spermine was greater than 0.5 mM. In general, a similar phenomenon to the case of K8 can be observed, while the crossing point from promotion to suppression now is about 0.04 mM. For example, the value of μ correspondingly changed from −1.5 (0.002 mM spermine) to −1.2 (spermine + 3 mM CaCl_2_), −0.68 (spermine + 3 mM MgCl_2_), respectively. The charge neutralization and inversion of DNA is promoted by adding di-valent counterions. On the other hand, the value of mobility changed correspondingly from 0.1 (1 mM spermine) to −0.46 (spermine + 3 mM CaCl_2_), −0.42 (spermine + 3 mM MgCl_2_), respectively. It is remarkable that the positive mobility can be switched back to a negative value by adding di-valent counterions. To further confirm our findings, we added di-valent ions to K4-DNA and cobalt hexamine-DNA solution to measure DNA electrophoresis mobility. The result are shown in Figure 3c,d. We can also see the promoting and suppressing effect for DNA charge compensation.

**Figure 4:**
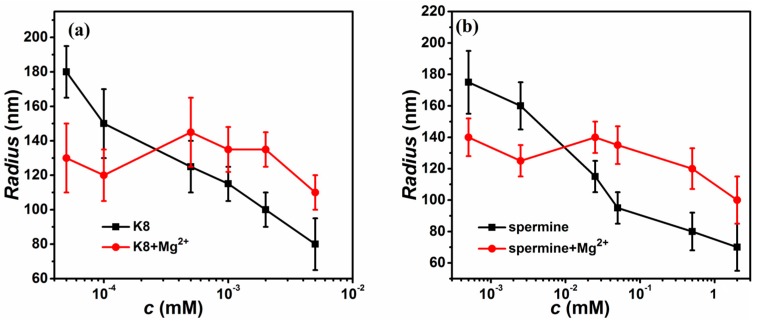
The hydrodynamic radius of condensed DNA as a function of the multivalent ions in a buffer containing di-valent ions (3 mM Mg^2+^). (**a**) The data for K8, K8-Mg^2+^ solution (black, red). (**b**) The data for spermine, spermine-Mg^2+^ solution (black, red).

When additional Mg^2+^ was added to the solution, the hydrodynamic radius of DNA changed accordingly. We plotted DNA hydrodynamic radius as a function of the multivalent ions (K8, spermine) in a buffer containing di-valent ions (3 mM Mg^2+^) in Figure 4. We can see that hydrodynamic radius of lambda DNA goes down from about 185 to 80 nm with increasing K8 concentration in absence of Mg^2+^ as shown in Figure 4a. While an additional 3 mM Mg^2+^ was added to the solution, the hydrodynamic radius of DNA varied in a way similar to its mobility. More specifically, the hydrodynamic radius decreases after mixing when K8 concentration is less than about 0.0002 mM. For example, the hydrodynamic radius of lambda DNA is about 185 nm at 0.00005 mM of K8 concentration, and it decrease to about 130 nm when Mg^2+^ of 3 mM is added to the solution. However, the mixing effect is switched to the opposite side when K8 concentration is larger than 0.0002 mM. For example, the hydrodynamic radius of lambda DNA is about 100 nm at 0.002 mM of K8 concentration, and it increases to about 135 nm when Mg^2+^ of 3 mM is added to the solution. Thus, DNA condensation is suppressed by mixing K8 and di-valent ions in that range. The result is also similar in the case of spermine, shown in Figure 4b.

**Figure 5:**
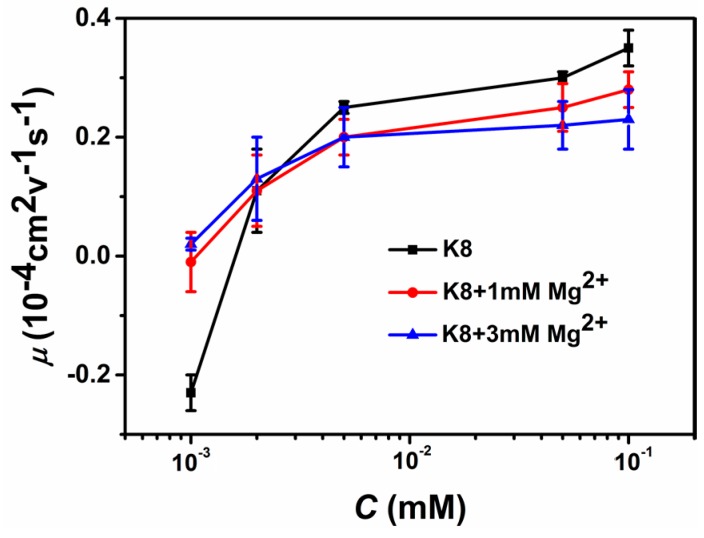
The comparison of electrophoretic mobility of DNA with di-valent ions (1 mM Mg^2+^, 3 mM Mg^2+^) in 1 mM TRIS solution.

The concentration of mono- or di-valent counterions might influence the variation of electrophoretic mobility and DNA compaction. We did a control experiment by lowering Mg^2+^ concentration to 1 mM in K8 solution. The comparison of electrophoresis mobility is shown in Figure 5, which is very similar to the case of 3 mM di-valent counterions but with a slightly weaker effect.

The suppression or inhibition on DNA compaction or charge neutralization was also observed by different experimental approaches. For example, Yoshikawa’s group observed the reversing effect of di-valent on the higher-order structure of giant DNA by fluorescence microscopy. It was found that di-valent cations, Mg^2+^ and Ca^2+^, inhibit DNA compaction induced by a trivalent cation, spermidine [44]. In a similar way, the charge neutralization and inversion of DNA by multivalent counterion is suppressed when adding di-valent counterions at normal ionic strength. However, the present measurement of electrophoretic mobility shows that under low ionic strength (<0.00064 for K8) charge neutralization of DNA induced by the multivalent counterion is promoted when adding extra mono- and di-valent counterions. The finding makes the picture of the mixing effect of counterions on DNA complete. 

### 3.2. Condensing Force and Unraveling Force Measurement of DNA Complex

DNA compaction is the process of one or few DNA molecular going from free state to a more compactly ordered structure to overcome the electrostatic repulsion between segments of DNA [9,10]. The DNA compaction involves of like-charge attraction and related charge compensation or neutralization. The latter plays a significant role in various types of macromolecular organization in charged systems [27]. For DNA system, like-charge attraction leads to DNA compaction, in which condensing force is closely related to its charge neutralization. In previous section, we measured the electrophoretic mobility of DNA in solutions of low ionic strength, and found the promotion effect of mixing counterion on charge neutralization. Now we try to find out whether the same effect is valid on DNA compaction by pulling DNA-condensing agent complexes in a flow cell with a home-made magnetic tweezers. In the setup, we can see the tethered DNA compaction and measure the tethering force simultaneously when adding a condensing agent.

**Figure 6:**
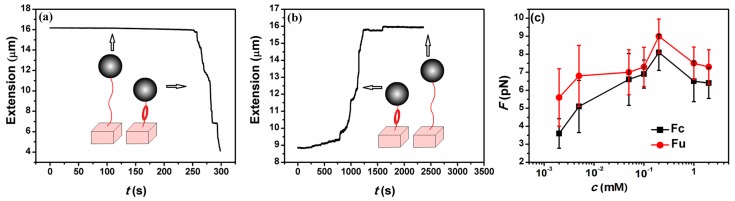
The curve of condensing and unraveling forces. (**a**) DNA extension-time curve measured by MT in DNA compaction process with K4. (**b**) DNA extension-time curve measured by MT in DNA pulling process with K4. (**c**) *F*_C_ and *F*_U_ of DNA in K4 solution.

DNA compaction causes a rapid step-like decrease in its extension when magnetic force (*F)* decreases below the condensation force, *F*c. In general, this shrinkage of DNA length is thought to be initiated by the spontaneous nucleation of a loop in the DNA [36]. Figure 6 presents typical extension-time curves of DNA compaction induced by K4, in which (a) shows a condensing curve while (b) denotes a unraveling curve of DNA. The condensing force and unravelling curves are shown in Figure 6c with increasing K4 concentration. From the black curve of *F*c, we can see that the condensing force initially increases rapidly and then reaches its maximal value around 0.2 mM K4 concentration, and finally decreases. The peak of force usually corresponds to the state of DNA with most neutralized charge since the electrostatic repulsive force is minimized in the state. When the concentration of K4 goes up further, the DNA charge might be over-compensated, resulting in the decrease of condensing force because of the regained Columbic repulsion between segments of DNA [51]. The red curve of unraveling force *F*_U_ in Figure 6c shows the same trend as the condensing force *F*c but its value is bigger than *F*c at the corresponding concentration of K4. It is understandable, since the unraveling process might involve the overlapping of different segments of DNA after condensation. In general, the maximal condensing or unraveling force corresponds to the complete neutralization of DNA charge, both under- or over-compensation of charge results in the decreasing force due to the charge depending Columbic repulsion.

**Figure 7:**
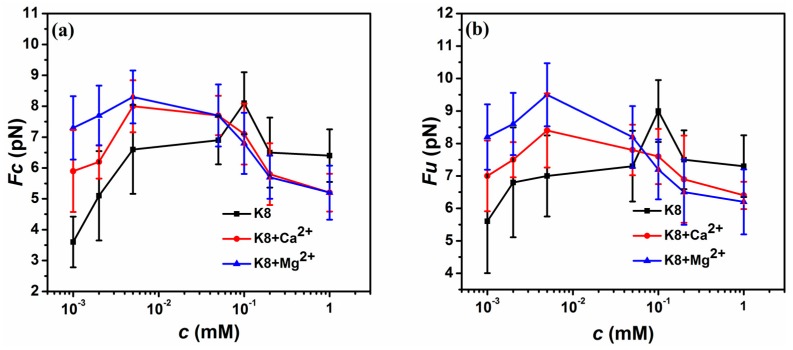
*F*_C_ and *F*_U_ of DNA in various solvent condition measured by MT. (**a**) *F*_C_ of DNA in different concentration of K8 (black square) or K8 + 3 mM CaCl_2_ (red point) or K8 + 3 mM MgCl_2_ (blue triangle). (**b**) *F*_U_ of DNA in different concentration of K8 (black square) or K8 + 3 mM CaCl_2_ (red point) or K8 + 3 mM MgCl_2_ (blue triangle).

Figure 7 shows measurements of the condensation force, *F*_C_ and unraveling force *F*_U_ as a function of the K8 concentration with mixing different di-valent cations. The black curve in Figure 7a exhibits an increase in *F*_C_ with increasing K8 concentration up to a maximum at about *c* = 0.1 mM, followed by a gradual decrease of *F*_C_ with further increasing concentration of K8. The red curve in Figure 7a denotes *F*_C_ as a function of K8 concentration when mixing 3 mM CaCl_2_ in solution, which shows the same trend as the case of only K8 in solution. The difference between them can be noted in the region of low K8 concentration, where *F*_C_ of tethering DNA is promoted in the presence of CaCl_2_ in solution. It means that the charge compensation of DNA by multivalent counterion is promoted when adding extra di-valent counterions in the region. This phenomenon is quite different from the case we have observed at mild spermine concentration (>0.4 mM) [42]. The blue curve in Figure 7a denotes *F*_C_ as a function of the K8 concentration when 3 mM MgCl_2_ is added in the solution. It shows a similar trend to the condensing force curve of CaCl_2_, but with bigger condensing force at the region of very low concentration of K8. From the whole interval, we can see that magnesium ions show more significant promotion effects, but the suppression effect has a little difference and it is difficult to distinguish between MgCl_2_ and CaCl_2_. Figure 7b denotes *F*_U_ as a function of the K8 concentration in different di-valent cations. The unraveling curves show the same trend with *F*_C_ but with a bigger value at its corresponding concentration. In the unraveling procedure, we can see more obvious promotion and suppression effect. In the same way, MgCl_2_ solution shows more significant influence on the promotion effect compared with CaCl_2,_ and the suppression effect has a little difference.

## 4. Discussion and Conclusions

When more than one counterion exists in solution, the electrostatic interaction between DNA and counterions becomes more complicated due to their cooperative and competitive interactions. For example, it has been shown that monovalent salt of high concentration always has an inhibitory effect on protamine-induced compaction [47]. Meanwhile, the competition between monovalent and multivalent ions results in that the condensation of polyelectrolyte is quite sensitive to ion-specific short-range interactions [45]. On the other hand, the increasing monovalent salt concentration has a negative impact on the charge neutralization by multivalent ions [48]. By statistical mechanics and electrostatics in solution, we can propose a microscopic mechanism of present experimental observation. When the concentration of mono- or di-valent cations is high in the bulk solution, the interaction between DNA and multivalent counterions is weakened. This leads to the effect of the release of multivalent counterions into the bulk solution, accompanied by the association between DNA and the oppositely charged mono- or di-valent cations. Thus, the increase in translational entropy upon the release of the multivalent counterions results in the decrease of charge compensation of DNA-complex. In the view of electrostatics, this mixing effect of counterions is due to electric shielding by small counterions. Thus, this enhanced shielding at high salt concentrations shifts the binding equilibrium between DNA and multivalent counterions. The combination of change in the translational entropy of counterions and the shielding effect leads to the same weakening of the association of DNA and multivalent cations. However, the scenario changes dramatically because of the electrostatic instability of DNA complexed with multivalent cations like K8. In the region of low concentration, the polycation do not compensate the main part of DNA charge, resulting in the effect of the surviving electric charge along the DNA chain. In other words, the polycations bind to the DNA chain with a relatively large spacing between neighboring counterions because of their highly charged features. Thus, there are many negative charges to some extent in the spacings. The surviving electric charge of DNA is further neutralized by the mono- or di-valent cations in solution. Thus, we can expect a promotion effect at a low concentration of multivalent counterions and a suppression effect at a higher concentration of multivalent counterions. In other words, if the concentration of counterions is lower than the critical concentration, DNA charge neutralization is incomplete. The mono and di-valent counterions can neutralize the remaining part of DNA compensated by multivalent ions. Thus, this mixing effect can be attributed to the cooperative electrostatic binding of counterions to DNA. The present argument is tentative and qualitative to provide a reasonable physical picture. We expect a molecular dynamics simulation of the system to give more insight for a quantitative explanation in future.

In summary, we have used DLS to demonstrate that the electrophoretic mobility of DNA in multivalent counterion solution (octalysine, spermine) increases in the presence of mono- and di-valent ions under the condition of low ionic strength in solution. This finding means that the charge neutralization of DNA by the multivalent counterion is promoted rather than suppressed when adding extra mono- and di-valent counterions in the ionic environment. This conclusion is also supported by the measurement of condensing and unraveling forces of DNA condensates induced by mixing counterions at low ionic strength by single molecular MT. We attributed the suppression effect to the cooperative electrostatic binding of counterions to DNA when the concentration of counterions in solution is below a critical concentration.

## References

[1] Cherstvy A.G. (2011). Electrostatic interactions in biological DNA-related systems. Phys. Chem. Chem. Phys..

[2] Thomas T.J., Tajmirriahi H.A. (2016). Polyamine-DNA interactions and development of gene delivery vehicles. Amino Acids.

[3] Thomas M., Klibanov A.M. (2003). Non-viral gene therapy: Polycation-mediated DNA delivery. Appl. Microbiol. Biotechnol..

[4] Yin H., Kanasty R.L., Eltoukhy A., Vegas A.J., Dorkin J.R., Anderson D.G. (2014). Non-viral vectors for gene-based therapy. Nat. Rev. Genet..

[5] Todd B.A., Rau D.C. (2008). Interplay of ion binding and attraction in DNA condensed by multivalent cations. Nucleic Acids Res..

[6] Yang J., Rau D.C. (2005). Incomplete Ion Dissociation Underlies the Weakened Attraction between DNA Helices at High Spermidine Concentrations. Biophys. J..

[7] Bloomfield V. (1996). DNA condensation. Curr. Opin. Struct. Biol..

[8] Zhou J., Ke F., Liang D. (2010). Kinetic study on the reentrant condensation of oligonucleotide in trivalent salt solution. J. Phys. Chem. B.

[9] Bloomfield V.A. (1997). DNA condensation by multivalent cations. Biopolymers.

[10] Besteman K., Van Eijk K., Lemay S. (2007). Charge inversion accompanies DNA condensation by multivalent ions. Nat. Phys..

[11] Luan B., Aksimentiev A. (2010). Electric and Electrophoretic Inversion of the DNA Charge in Multivalent Electrolytes. Soft Matter.

[12] Besteman K., Zevenbergen M.A., Heering H.A., Lemay S.G. (2004). Direct observation of charge inversion by multivalent ions as a universal electrostatic phenomenon. Phys. Rev. Lett..

[13] Todd B.A., Parsegian V.A., Shirahata A., Thomas T., Rau D.C. (2008). Attractive forces between cation condensed DNA double helices. Biophys. J..

[14] Rau D.C., Parsegian V.A. (1992). Direct measurement of the intermolecular forces between counterion-condensed DNA double helices. Evidence for long range attractive hydration forces. Biophys. J..

[15] Yoo J., Aksimentiev A. (2016). The structure and intermolecular forces of DNA condensates. Nucleic Acids Res..

[16] Allahyarov E., Gompper G., Lowen H. (2004). Attraction between DNA molecules mediated by multivalent ions. Phys. Rev. E.

[17] Buyukdagli S., Blossey R. (2016). Correlation-induced DNA adsorption on like-charged membranes. Phys. Rev. E.

[18] Buyukdagli S., Ala-Nissila T. (2017). Multivalent cation induced attraction of anionic polymers by like-charged pores. J. Chem. Phys..

[19] Wang Y., Ran S., Man B., Yang G. (2011). Ethanol induces condensation of single DNA molecules. Soft Matter.

[20] Wang Y., Ran S., Man B., Yang G. (2011). DNA condensations on mica surfaces induced collaboratively by alcohol and hexammine cobalt. Colloids Surf. B Biointerfaces.

[21] Qiu S., Wang Y., Cao B., Guo Z., Chen Y., Yang G. (2015). The suppression and promotion of DNA charge inversion by mixing counterions. Soft Matter.

[22] Xia W., Wang Y., Yang A., Yang G. (2017). DNA Compaction and Charge Inversion Induced by Organic Monovalent Ions. Polymers.

[23] Guo Z., Wang Y., Yang A., Yang G. (2016). The effect of pH on charge inversion and condensation of DNA. Soft Matter.

[24] Wang Y., Wang R., Cao B., Guo Z., Yang A., Yang G. (2016). Single molecular demonstration of modulating charge inversion of DNA. Sci. Rep..

[25] Arscott P.G., Ma C., Wenner J.R., Bloomfield V.A. (1995). DNA condensation by cobalt hexaammine (III) in alcohol-water mixtures: Dielectric constant and other solvent effects. Biopolymers.

[26] Mao W., Gao Q., Liu Y., Fan Y., Hu L., Xu H. (2016). Temperature dependence of DNA condensation at high ionic concentration. Mod. Phys. Lett. B.

[27] Baigl D., Yoshikawa K. (2005). Dielectric control of counterion-induced single-chain folding transition of DNA. Biophys. J..

[28] Li C., Ma C., Xu P., Gao Y., Zhang J., Qiao R., Zhao Y. (2013). Effective and Reversible DNA Condensation Induced by a Simple Cyclic/Rigid Polyamine Containing Carbonyl Moiety. J. Phys. Chem. B.

[29] Lee J.H., Amin R., Kim B., Ahn S.J., Lee K.W., Kim H.J., Park S.H. (2012). The restoration of DNA structures by the dry-wet method. Soft Matter.

[30] Oda Y., Sadakane K., Yoshikawa Y., Imanaka T., Takiguchi K., Hayashi M., Kenmotsu T., Yoshikawa K. (2016). Highly Concentrated Ethanol Solutions: Good Solvents for DNA as Revealed by Single-Molecule Observation. ChemPhysChem.

[31] Amaduzzi F., Bomboi F., Bonincontro A., Bordi F., Casciardi S., Chronopoulou L., Diociaiuti M., Mura F., Palocci C., Sennato S. (2014). Chitosan-DNA complexes: Charge inversion and DNA condensation. Colloids Surf. B Biointerfaces.

[32] Dai L., Jones J.J., Klotz A.R., Levy S., Doyle P.S. (2017). Nanoconfinement greatly speeds up the nucleation and the annealing in single-DNA collapse. Soft Matter.

[33] Pecora R. (1985). Dynamic Light Scattering: Applications of Photon Correlation Spectroscopy.

[34] Mengarelli V., Auvray L., Pastre D., Zeghal M. (2011). Charge inversion, condensation and decondensation of DNA and polystyrene sulfonate by polyethylenimine. Eur. Phys. J. E Soft Matter.

[35] Dias R.S., Innerlohinger J., Glatter O., Miguel M.G., Lindman B. (2005). Coil-Globule Transition of DNA Molecules Induced by Cationic Surfactants: A Dynamic Light Scattering Study. J. Phys. Chem. B.

[36] Fu W., Wang X., Zhang X., Ran S., Yan J., Li M. (2006). Compaction dynamics of single DNA molecules under tension. J. Am. Chem. Soc..

[37] Yan J., Skoko D., Marko J.F. (2004). Near-field-magnetic-tweezer manipulation of single DNA molecules. Phys. Rev. E.

[38] Hinterdorfer P., Dufrene Y.F. (2006). Detection and localization of single molecular recognition events using atomic force microscopy. Nat. Methods.

[39] Neuman K.C., Nagy A. (2008). Single-molecule force spectroscopy: Optical tweezers, magnetic tweezers and atomic force microscopy. Nat. Methods.

[40] Japaridze A., Benke A., Renevey S., Benadiba C., Dietler G. (2015). Influence of DNA Binding Dyes on Bare DNA Structure Studied with Atomic Force Microscopy. Macromolecules.

[41] Manning G.S. (1969). Limiting Laws and Counterion Condensation in Polyelectrolyte Solutions I. Colligative Properties. J. Chem. Phys..

[42] Qiu X., Giannini J., Howell S.C., Xia Q., Ke F., Andresen K. (2013). Ion Competition in Condensed DNA Arrays in the Attractive Regime. Biophys. J..

[43] Yoo J., Aksimentiev A. (2012). Competitive binding of cations to duplex DNA revealed through molecular dynamics simulations. J. Phys. Chem. B.

[44] Tongu C., Kenmotsu T., Yoshikawa Y., Zinchenko A.A., Chen N., Yoshikawa K. (2016). Di-valent cation shrinks DNA but inhibits its compaction with trivalent cation. J. Chem. Phys..

[45] Burak Y., Ariel G., Andelman D. (2004). Competition between condensation of monovalent and multivalent ions in DNA aggregation. Curr. Opin. Colloid Interface Sci..

[46] Makita N., Ullner M., Yoshikawa K. (2006). Conformational change of giant DNA with added salt as revealed by single molecular observation. Macromolecules.

[47] Makita N., Yoshikawa Y., Takenaka Y., Sakaue T., Suzuki M., Watanabe C., Kanai T., Kanbe T., Imanaka T., Yoshikawa K. (2011). Salt Has a Biphasic Effect on the Higher-Order Structure of a DNA−Protamine Complex. J. Phys. Chem. B.

[48] Van der Heyden F.H., Stein D., Besteman K., Lemay S.G., Dekker C. (2006). Charge inversion at high ionic strength studied by streaming currents. Phys. Rev. Lett..

[49] Tanaka M. (2004). Electrophoresis of a rod macroion under polyelectrolyte salt: Is mobility reversed for DNA?. J. Phys. Condens. Matter.

[50] Tanaka M., Grosberg A.Y. (2002). Electrophoresis of a charge-inverted macroion complex: Molecular-dynamics study. Eur. Phys. J. E.

[51] Murayama Y., Sakamaki Y., Sano M. (2003). Elastic response of single DNA molecules exhibits a reentrant collapsing transition. Phys. Rev. Lett..

